# Prednisolone-induced virginal mammary hypertrophy: Case report

**DOI:** 10.1016/j.ijscr.2019.04.042

**Published:** 2019-05-16

**Authors:** Samir Jabaiti, Luma Fayyad, Ula Isleem

**Affiliations:** a Department of Plastic and Reconstructive surgery, Jordan University Hospital, Amman, Jordan; b Department of Pathology, Jordan University Hospital, Amman, Jordan; c Department of Pathology, King Hussein Medical Center, Amman, Jordan; d Faculty of Medicine, University of Jordan, Amman, Jordan

**Keywords:** Virginal mammary hypertrophy, Prednisolone, Mammaplasty, Case report

## Abstract

**Introduction:**

Virginal mammary hypertrophy (VMH) is a rare benign disorder of the breast characterized by excessive enlargement of one or both breasts. It usually presents during adolescence. Drug-induced VMH has been scarcely reported in case reports. Review of the literature showed that prednisolone-induced gigantomastia was reported in a single study on a 47-year-old female. In this study, a case of VMH in a 17-year-old girl following prednisolone treatment will be described. Clinical presentation, clinical findings, diagnostic work-up, management and follow up are discussed.

**Presentation of case:**

A 17-year-old, single female presented to the plastic surgery department at our institute with progressive enlargement of both breasts, 2 months following treatment with prednisolone. Incisional biopsy excluded other breast differential diagnoses. The patient was managed surgically with bilateral mammaplasty with free nipple-areola graft. A total of 8.325 kg of breast tissue was resected. Follow-up 48 months postoperatively revealed good patient satisfaction with no recurrence of breast hypertrophy.

**Discussion:**

Although the estrogen theory is regarded as the most credible explanation for VMH, several cases of drug-induced mammary hypertrophy have been reported. This study may be the first reported case of adolescent prednisolone-induced VMH. Mammaplasty is an accepted treatment despite its possible association with a higher recurrence rate.

**Conclusion:**

VMH may be a rare complication of prednisolone treatment. It should be considered in patients presenting with breast hypertrophy following steroid administration.

## Highlights

• Virginal Mammary Hypertrophy is a rare condition.
• Virginal Mammary Hypertrophy can be physically and psychologically debilitating.
• A 17-year old with breast hypertrophy following prednisolone treatment is discussed.
• The patient was treated with a bilateral mammaplasty following appropriate counseling.
• Four years after the original procedure, there was no recurrence of hypertrophy.

## 1 Introduction

This case report has been reported in accordance with the SCARE criteria [1].

Virginal mammary hypertrophy (VMH) is a benign disorder of the breast characterized by excessive enlargement of one or both breasts, which usually presents during adolescence. This condition may cause physical and psychological disability to patients at a critical stage of their life [2]. A variety of names have been given to this pathology in literature: juvenile hypertrophy, virginal/ juvenile macromastia, gigantomastia, juvenile gigantomastia, virginal breast hypertrophy, and macromastia [3].

There is no universal consensus on the definition of this pathology. Dafydd et al. defined gigantomastia as excess breast tissue that contributes 3% or more to the patient's total body weight [4].

The etiology of VMH has not yet been elucidated [3]. The estrogen theory discussed by Griffith is still regarded as the most credible explanation for abnormal breast enlargement. The estrogen theory includes excess local estrogen production within the breast tissue, enhanced estrogen receptor sensitivity to normal levels of estrogen, and the presence of an estrogen-like substance that mimics the effects of estrogen-producing ductal proliferation, or any combination of these factors [5]. This theory was supported by reports revealing increased estrogen receptor activity in the resected breast tissue [6], although other studies show normal receptor activity [7].

However, drug-induced mammary hypertrophy has been increasingly reported in a number of case reports. VMH has been associated with certain medications, such as neothetazone, cyclosporine, D-penicillamine, bucillamine, and propylthiouracil [[8], [9], [10], [11], [12]].

Drug-induced mammary hypertrophy secondary to steroid use was reported only once by Troccola et al who described a case of gigantomastia in a 47- year- old patient, in the setting of ovarian cancer, treated with cycles of chemotherapy combined with prednisolone [2]. In the following report, a case of juvenile mammary hypertrophy in a 17-year-old girl following prednisolone treatment for mixed connective tissue disease is presented.

## 2 Presentation of case

In August of 2014, a 17-year-old female patient was referred to the Plastic Surgery Department of our institute. The patient complained of progressive enlargement of both breasts over a period of 14 months. Upon presentation to the outpatient clinic, the patient also complained of neck, shoulder, and back pain that caused marked limitation of their daily activities, as well as significant social embarrassment. The patient began menarche at 12 years of age. The patient denied a family history of similar breast disorders or of breast cancer.

Sixteen months prior to presentation to our hospital, the patient presented to another hospital complaining of diffuse joint pain, malar rash, skin rash, chest pain, digital swelling, and Raynaud's phenomena. The patient’s work-up then showed anemia, neutropenia, and a positive ANA serology. The patient was diagnosed as a case of mixed connective tissue disease.

The patient was then started on a treatment course of 16 mg of oral prednisolone twice daily for eight months. The medication was then tapered to 10 mg daily for another six months. The enlargement began two months after initiating treatment, however, her treatment was maintained. The patient did not receive penicillamine, bucillamine, cyclosporine, or any other related drugs. When the patient presented to our hospital, they had undergone improvement in her symptoms.

**Fig. 1:**
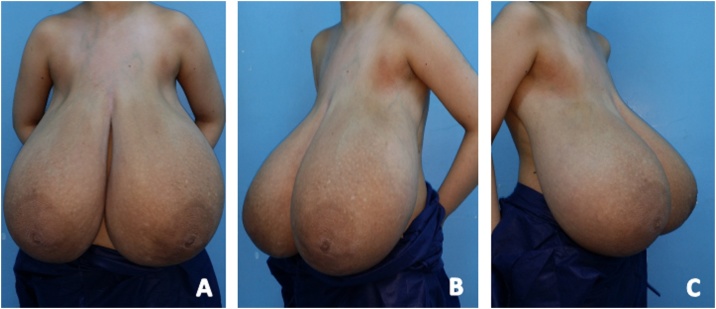
(A) Anterior view of a 17-year old with bilateral mammary hypertrophy (B) Left lateral view (C) Right lateral view.

On clinical examination, the patient's weight was 75 kg, height was 1.60 m, and body mass index (BMI) was calculated as 29.3 kg /m2. Breast examination showed massive bilateral enlargement with marked ptosis, widened areolas, and dilated subcutaneous veins. Shoulder grooving from brassiere straps and intertrigo at the inframammary folds were also noted. (Fig. 1: (A) Anterior view of a 17-year old with bilateral mammary hypertrophy (B) Left lateral view (C) Right lateral view) Palpation revealed firm breast texture with diffuse nodularity. There was no axillary lymphadenopathy.

Routine biochemical examinations and endocrine investigations were within normal limits. The patient's hormonal workup included LH, FSH, prolactin, progestin, 17-estradiol, T3, T4, TSH, and cortisol. Breast CT scan showed both breasts were hugely enlarged with dense parenchymal tissue bilaterally, associated with dependent fluid accumulation.

Incisional biopsy was performed to exclude other breast pathologies, such as virginal fibroadenoma, fibrocystic disease, phyllodes tumor, and breast lymphoma. It showed markedly increased collagenization of the stroma with areas of pseudoangiomatous stromal hyperplasia (PASH). The glandular component showed focal adenosis and foci of fibroadenomatoid hyperplasia were also seen.

The patient and her family were counseled regarding surgical options for treatment of the patient's condition, namely, subcutaneous mastectomy versus bilateral mammaplasty. They were informed of the likelihood of higher recurrence, yet a more desirable cosmetic outcome of the second option. They were also informed of possible complications, as well as the possibility of additional interventions that may be required in the future.

Bilateral breast reduction with nipple-areola complex transfer as a free graft was performed under general endotracheal intubation. A total of 8.325 kg of breast tissue was resected. (4225 g from the right side and 4100 g from the left side). The patient had an uneventful postoperative recovery with excellent graft take of the nipple-areola complex. The patient was discharged after 5 days and was followed up in the outpatient clinic.

**Fig. 2:**
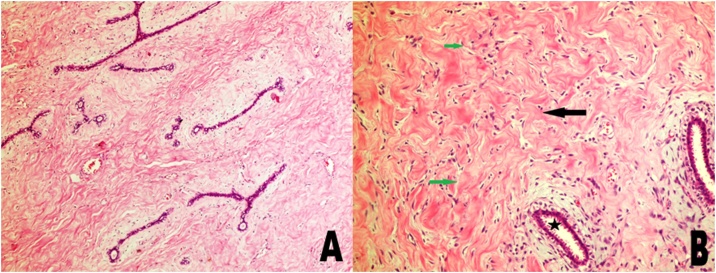
(A) Marked Collagenization and hyalinizing fibrosis of the breast stroma with compression of benign breast ducts into slit-like spaces in a fibroadenomatoid pattern. Hematoxylin and Eosin stain x40 (B) Higher power view; Black Arrow: Hyalinizing fibrosis of breast tissue stroma; Green Arrows: Pseudoangiomatous stromal hyperplasia; Star: Benign ductal epithelium. Hematoxylin and Eosin stain x100.

The final histopathology report showed similar features to those seen in the incisional biopsy; with additional findings in the form of patchy infiltration of the ductal epithelium by lymphocytes and concentric “onion skin” type fibrosis surrounding small blood vessels with perivascular lymphoplasmacytic infiltration were also seen. The findings were consistent with juvenile macromastia and lymphocytic inflammation. There was no evidence of in-situ or invasive malignancy, lymphoma, or vasculitis. (Fig. 2: (A) Marked Collagenization and hyalinizing fibrosis of the breast stroma with compression of benign breast ducts into slit-like spaces in a fibroadenomatoid pattern. Hematoxylin and Eosin stain x40 (B) Higher power view; Black Arrow: Hyalinizing fibrosis of breast tissue stroma; Green Arrows: Pseudoangiomatous stromal hyperplasia; Star: Benign ductal epithelium. Hematoxylin and Eosin stain x100)

**Fig. 3:**
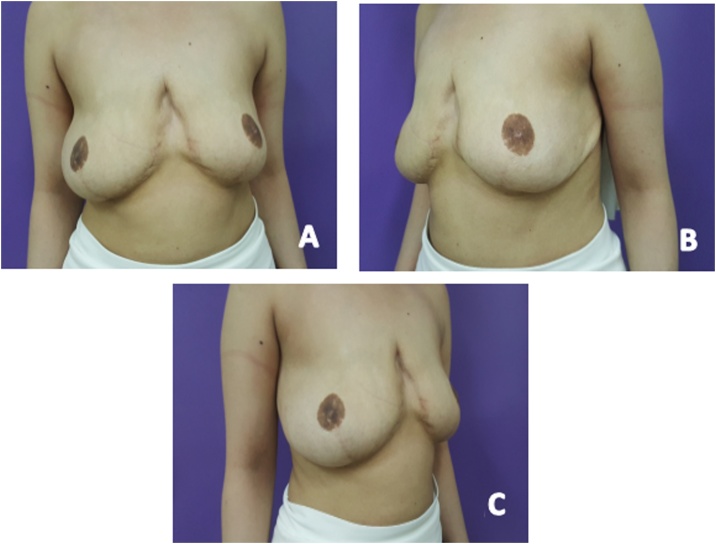
Anterior view post mammaplasty at 34 months follow-up (B) Left lateral view (C) Right lateral view.

The patient did not receive any hormonal treatment, neither preoperatively or postoperatively. Twenty months after breast reduction, the patient underwent a scar revision procedure. Forty-eight months after the original procedure, the patient was found to be satisfied and there was no recurrence of breast hypertrophy. (Fig. 3: (A) Anterior view post mammaplasty at 34 months follow-up (B) Left lateral view (C) Right lateral view)

## 3 Discussion

Virginal mammary hypertrophy is a rare condition. It has been reported sporadically in medical literature with no prospective or observational trials [3]. Adolescent females suffering from this debilitating condition are vulnerable to developing a negative body image and significant psychological stresses. In addition, social issues arise secondary to poor fitting clothing, trouble exercising, and public scrutiny as a result of their enlarged breasts [13]. Furthermore, there are physical complications including back, neck and shoulder pains, as well as intertrigo at the inframammary folds [14].

Drug-induced mammary hypertrophy has been reported in scattered case reports in the literature. Scott reported a possible relation between the antibiotic Neothetazone and the gigantomastia [8]. Penicillamine is the drug most commonly reported as an etiological factor in mammary hypertrophy, however, the mechanism of action is poorly understood [10]. Sakai et al reported the first case of bucillamine-induced giant mammary hyperplasia in a 25-year-old woman who was treated for rheumatoid arthritis. The authors believed that bucillamine was the cause of the giant hypertrophy because of its structural similarity to D-penicillamine [11]. O’Hare reported a case of a 12-year-old girl who developed bilateral mammary hypertrophy four months after initiation of propylthiouracil for the treatment of thyrotoxicosis [12].

A literature review revealed that prednisolone induced gigantomastia was reported only once by Troccola et al in 2011 [2]. The report described a case of gigantomastia in a 47 -year-old woman within 2 months of starting adjuvant chemotherapy protocol, including prednisolone, for ovarian cancer. The patient was treated with a bilateral breast reduction. The authors believed that prednisolone was implicated in gigantomastia since the same patient did not develop breast hypertrophy when treated with the adjunctive chemotherapy protocol without prednisolone for the treatment of cancer recurrence 2 years later. Our case report may support the correlation between prednisolone and breast hypertrophy.

Treatment of VMH is challenging. There are no accepted, evidence-based guidelines for its management due to the scarcity of reported cases and the absence of prospective or observational trials regarding the condition. Surgical treatment, medical treatment, or a combination of both have been attempted to manage this pathology. The two main surgical options discussed include reduction mammaplasty as a pedicle-based procedure or with free nipple graft, the second option is mastectomy with immediate or delayed breast reconstruction [3].

Reduction mammaplasty is a safe and effective treatment that can greatly alleviate the social, psychological, and physical strain caused by macromastia in adolescents [14]. Moreover, reduction mammaplasty has been associated with good overall satisfaction and improvements in quality of life on long-term follow-up [13].

The drawback of reduction mammaplasty is its higher reported recurrence rate compared with subcutaneous mastectomy, as shown in a meta-analysis of the case reports conducted by Hoppe et al. in 2011 [3]. Nevertheless, considering the depressing psychological consequences and negative cosmetic outcome of mastectomy on these adolescent patients, reduction mammaplasty is still the most popular approach to manage virginal mammary hypertrophy [3,13].

Many drugs, including several hormone-modulators, have been reported either alone or combined with surgery to manage VMH. Dydrogesterone (a progesterone analog) and tamoxifen (selective estrogen receptor modulator) prove to be the most popular choices. Tamoxifen has been used preoperatively to arrest breast growth and postoperatively to decrease recurrence with a variable success rate. However, at this time, there is inadequate reported experience with medical therapy for adolescents with juvenile breast hypertrophy to predict risks and benefits [15].

Furthermore, Tamoxifen has been associated with serious side effects that should be considered, especially in the younger population that suffers from virginal mammary hypertrophy. Side effects include endometrial hyperplasia—increasing the risk of endometrial cancer—hot flashes, increased risk of venous thrombosis, and bone density changes [16]. Taking into consideration these potential side effects of Tamoxifen versus its unverified benefits, it was not used preoperatively or postoperatively in our patient.

## 4 Conclusion

VMH is a very rare complication of prednisolone treatment. This correlation should be considered in patients presenting with breast hypertrophy while on steroids or related medications. Reduction mammaplasty is a reasonable option of surgical treatment owing to its positive psychological and cosmetic benefits. However, the patient and their family should be informed regarding the higher likelihood of recurrence.

## Conflicts of interest

There is no conflict of interest to be declared.

## Funding

This research did not receive any specific grant from funding agencies in the public, commercial, or not-for-profit sectors.

## Ethical approval

The study was approved by the IRB of our institute.

## Consent

Written informed consent was obtained from the patient for publication of this case report and the accompanying images. All identifying details have been omitted. A copy of the written consent is available for review by the Editor-in-Chief of this journal on request.

## Author contribution

Samir Jabaiti → Conceptualization, supervision, and data curation, as well as writing –Original draft

Luma Fayyad → Data curation, as well as writing-original draft

Ula Isleem → Writing – Review and Editing and Project administration

## Registration of research studies

NA.

## Guarantor

Samir K Jabaiti

Ula N Isleem

## Provenance and peer review

Not commissioned, externally peer-reviewed.

## References

[1] Agha RA, Borrelli MR, Farwana R, Koshy K, Fowler A, Orgill DP, For the SCARE Group. The SCARE 2018 Statement: Updating Consensus Surgical CAse REport (SCARE) Guidelines, International Journal of Surgery 2018;60:132-136 10.1016/j.ijsu.2018.10.028 30342279

[2] Troccola A, Maruccia M, Dessy LA, Onesti MG. Cortisone-induced gigantomastia during chemotherapy, G. Chir. 2011;32;266-9. 21619780

[3] Hoppe IC, Patel PP, Singer-Granick CJ, Granick MS. Virginal mammary hypertrophy: a meta-analysis and treatment algorithm,Plast Reconstr Surg. 127 2011; 2224-31. doi:10.1097/PRS.0b013e3182131bd1. 10.1097/PRS.0b013e3182131bd1 21617457

[4] Dafydd H, Roehl KR, Phillips LG, Dancey A, Peart F, Shokrollahi K. Redefining gigantomastia, J. Plast. Reconstr. Aesthet. Surg. 2011;64;160-3. doi: 10.1016/j.bjps.2010.04.043. 10.1016/j.bjps.2010.04.043 20965141

[5] Griffith JR. Virginal breast hypertrophy, J. Adolesc. Health.Care. 1989;10;423–432. 10.1016/0197-0070(89)90224-6 2681107

[6] Noczyńska A, Wasikowa R, Myczkowski T. Hypersensitivity of estrogen receptors as a cause of gigantomastia in two girls (in Polish), Pol. Merkur. Lekarski. 2001;11;507–9. 11899849

[7] Gliosci A, Presutti F. Virginal gigantomastia: Validity of combined surgical and hormonal treatments, Aesthetic. Plast. Surg. 1993;17; 61–5 10.1007/BF00455051 8430532

[8] Scott EH. Hypertrophy of the breast, possibly related to medication: a case report, S. Afr. Med. J. 1970;44;449-50. 4192067

[9] Cerveli V, Orlando G, Giudiceandre F, Grimaldi M, Pisani F, Strati F, Iaria G, Piccione E, Torri E, Carluccio C, Tisone G, Casciani CU. Gigantomastia and breast lumps in a kidney transplant recipient, Transplantation Proc. 1999;31; 3224-5. 10.1016/s0041-1345(99)00701-0 10616452

[10] Desautels JE. Breast gigantism due to D-penicillamine, Can. Assoc. Radiol. J. 1994;45;143-4. 8149272

[11] Sakai Y, Wakamatsu S, Ono K, Kumagai N. Gigantomastia induced by bucillamine, Ann. Plast. Surg. 2002;49;193-5. 10.1097/00000637-200208000-00013 12187348

[12] O’Hare PM, Frieden IJ. Virginal breast hypertrophy, Pediatr. Dermatol. 2000;17;277–81. 10.1046/j.1525-1470.2000.01774.x 10990575

[13] Nguyen JT, H. Palladino, A.J. Sonnema, P.M. Petty. Long-term satisfaction of reduction mammaplasty for bilateral symptomatic macromastia in younger patients, J. Adolesc. Health. 2013;53;112-117. doi: 10.1016/j.jadohealth.2013.01.025. 10.1016/j.jadohealth.2013.01.025 23523309

[14] Wolfswinkel EM, Lemaine V, Weathers WM, Chike-Obi CJ, Xue AS, Heller L. Hyperplastic breast anomalies in the female adolescent breast, Semin. Plast. Surg. 2013; 27;49–55. doi: 10.1055/s-0033-1347167 10.1055/s-0033-1347167 PMC3706054 24872740

[15] Pruthi S, Jones KN. Nonsurgical management of fibroadenoma and virginal breast hypertrophy. Semin Plast Surg. 2013;62-6. doi: 10.1055/s-0033-1343997. 10.1055/s-0033-1343997 PMC3706058 24872742

[16] Koves IH, Zacharin M. Virginal breast hypertrophy of an 11-year-old girl. J Paediatr Child Health. 2007;4:315-7. 10.1111/j.1440-1754.2007.01067.x 17444838

